# Prevalence of smoking, alcohol consumption, and drug abuse in Iranian adults: Results of Azar Cohort Study

**DOI:** 10.34172/hpp.2023.12

**Published:** 2023-07-10

**Authors:** Sahar Naghizadeh, Elnaz Faramarzi, Hossein Akbari, Nasrin Jafari, Parvin Sarbakhsh, Asghar Mohammadpoorasl

**Affiliations:** ^1^Department of Statistics and Epidemiology, Faculty of Health, Tabriz University of Medical Sciences, Tabriz, Iran; ^2^Liver and Gastrointestinal Diseases Research Center, Tabriz University of Medical Sciences, Tabriz, Iran

**Keywords:** Tobacco smoking, Alcohol drinking, Health risk behaviors, Cohort studies

## Abstract

**Background:**

Substance abuse has significant health impacts on families and society as a whole. We aimed to provide detailed prevalence estimates of substance abuse among the Azar Cohort Study respondents in Tabriz, Iran.

**Methods:**

Data on 15006 participants of Azar Cohort Study were analyzed. The variables included tobacco smoking, alcohol use, drug abuse, and socio-demographic characteristics. The prevalence of substance abuse (with a 95% confidence interval) was calculated using the direct standardization method.

**Results:**

Overall, 9.3% and 6.2% of the participants were regular and heavy cigarette smokers, respectively. Also, 1.9% and 2.1% of participants reported a history of using illicit drugs and alcohol, respectively. Substance abuse was more prevalent among males than females. Substance abuse varied significantly with age and socioeconomic variables.

**Conclusion:**

We identified specific demographic and socioeconomic groups with a higher prevalence of all studied behaviors. Such high-risk groups should be targeted when designing substance abuse prevention programs.

## Introduction

 As one of the world’s most serious health concerns,^1^ substance abuse has major health impacts on addicts, their families, and society as a whole.^2^ According to the World Health Organization (WHO), smoking is the world’s second-leading cause of mortality,^3^ and alcohol use increases the risk of physical and mental disorders. Alcohol use also contributes to a variety of cancer-related mortalities and morbidities.^4,5^

 Tobacco and its derivatives are anticipated to kill more than six million individuals annually worldwide. Cigarette smoking is also expected to cause more than 8 million deaths yearly by 2030.^6^ Smoking is responsible for approximately 4% and 13% of years of living with disability in developed and developing countries, respectively.^7^ As half of all tobacco-associated deaths occur in middle-aged adults (35-61 years old), it can reduce the life expectancy of those in this age range by 20 to 25 years.^8^ A dose-response relationship between alcohol intake and 23 cancer types has been discovered; with an increase in alcohol use, a rise occurs in the risk of oral, pharyngeal, esophageal, colorectal, laryngeal, and breast cancers.^9^ On the other hand, drug abuse can lead to schizophrenia, psychosis, heart disease, asthma, pneumonia, and cognitive impairment.^10,11^

 The prevalence of smoking is roughly 22% worldwide^12^ and 17.4% in Iran.^13,14^ According to global data, the prevalence of drug abuse is rising, particularly in low and middle-income countries^15^ like Iran. Based on a recent Iranian study, 11.9% of the adult population reported taking illicit drugs at least once in their life.^14^

 In a previous study conducted by Zahirian et al, the prevalence of smoking among PERSIAN cohort study participants was 16.1%.^16^ In another Iranian study, the prevalence of smoking, alcohol use, and drug abuse were 14%, 9%, and 11%, respectively.^14^ In these studies, the participants were categorized into a maximum of three groups: smokers, non-smokers, and former smokers. Moreover, alcohol and drug abusers provided information about their history of usage only with yes/no questions, representing a source of unclarity about the exact prevalence of the behaviors in the Iranian setting. Therefore, more detailed prevalence estimates of smoking, alcohol use, and drug abuse seem necessary. Also, little evidence exists about substance abuse at the community level based on cohort studies. Our aim in the present study was to report detailed prevalence estimates of smoking, alcohol use, and drug abuse among the respondents of the Azar Cohort Study in Tabriz, Iran.

## Materials and Methods

###  Study setting

 The Azar Cohort Study is a part of the PERSIAN (the Prospective Epidemiological Research Studies in Iran) cohort study, which examines the risk factors of common non-communicable diseases among Iranian adults. In 2014, the Azar Cohort Study was established in Shabestar, a county in the East Azerbaijan province of Iran. The region covers an area of 2,630 km^2^, and its climate is influenced by its proximity to Lake Urmia. Its population is 124 499, with 48.5% residing in urban areas. Most of the population (68%) is aged between 15 and 64 years. Everyone is registered with the family physician (general practitioner) program. Individuals from 35 to 70 years of age were invited to participate in this study with the following inclusion criteria: permanent residence in the Shabestar district for at least nine months, written informed consent, and having at least one Azeri parent. The participants diagnosed with a disabling psychiatric and physical disorder were excluded from the study.^14,17,18^ In the present paper, we used data on 15,006 eligible participants of the Azar Cohort Study.

###  Measures

####  Tobacco smoking scale

 The tobacco smoking scale included five items: 1) If the respondents reported never smoking or smoking less than 100 cigarettes in their lifetime, they were defined as “non-smokers”; 2) If they reported that in their lifetime, they have either smoked more than 100 cigarettes or smoked one or more cigarettes a day, but have not smoked for at least one year, they were defined as “cigarette ex-smokers”; 3) If they reported that they currently smoke one or more cigarettes per day, they were defined as “regular cigarette smokers”; 4) If they announced that they currently smoke 20 and more cigarettes per day, they were defined as “heavy cigarette smokers”; 5) If they reported using other types of tobacco (such as pipe, hookah, etc) at least once a week, they were defined as “other types of tobacco users.”

####  Alcohol use scale

 A five-item scale was also used to assess alcohol use: 1) If respondents reported having never consumed alcohol in their lifetime, they were classified as “non-drinkers”; 2) If they noted to have consumed alcohol once/twice or less than 100 mL in their lifetime, they were defined as “experienced (1-2 times)”; 3) If they reported having consumed alcohol only for a limited period for treatment, they were defined as “limited time (for treatment)”; 4) If they reported that they had consumed alcohol more than twice in the past, and do not consume it currently, they were defined as “ex-drinkers”; 5) If they announced to have consumed alcohol recently (within 30 days), and more than twice in their lifetime, they were defined as “drinkers.”

####  Drug abuse scale

 This scale comprised three items: 1) If respondents noted to have never used illicit drug (such as heroin, amphetamines, barbiturates, cannabis, cocaine, hallucinogens, and opioids), they were classified as “non-drug abusers”; 2) If they reported having used illicit drugs once or twice in their lifetime, they were defined as “experienced (1-2 times)”; 3) If they reported having used the illicit drug more than twice in their lifetime, they were classified as “drug abusers.”

###  Socioeconomic status

 The participants’ socioeconomic status (SES) was defined based on job categories, educational level, and household assets using principal component analysis (PCA). SES was classified into very high, high, middle, low, and very low, based on a quintuple of the obtained scores.^18^

###  Statistical analysis

 Data are reported as frequency (percent). Due to the difference in the number of men and women, and the difference in the prevalence of substance abuse between the genders, the prevalence of substance abuse (with a 95% confidence interval) for the entire sample was calculated using the direct standardization method. The chi-squared test was used to assess the factors associated with substance abuse. Statistical analysis was performed using IBM SPSS Statistics for Windows version 21.0 (IBM Inc., Armonk, NY, USA), and statistical significance was set at 0.05, a priori.

## Results

 Out of 15 006 participants, 6,712 (44.7%) were males. Table 1 shows the frequency of substance abuse by gender. As depicted, 9.3% and 6.2% of the participants were regular and heavy cigarette smokers, respectively. Also, 1.9% and 2.1% of participants were illicit drug users and alcohol drinkers, respectively. Substance abuse was more prevalent among males than females.

**Table 1:** Prevalence of smoking, alcohol use, and drug abuse by gender (n = 15006).

**Stages **	**Male n (%)**	**Female n (%)**	**Total n (%)**	**Total standardized prevalence % (95% CI)**
Tobacco smoking				
Non-smoker	3205 (47.8)	8196 (98.8)	11401 (76.0)	73.2 (72.6-73.9)
Cigarette ex-smoker	1208 (18.0)	38 (0.5)	1246 (8.3)	9.2 (8.8-9.7)
Cigarette regular smoker	1217 (18.1)	32 (0.4)	1249 (8.3)	9.3 (8.8-9.7)
Heavy cigarette smoker	823 (12.3)	7 (0.1)	830 (5.5)	6.2 (5.8-6.6)
Using other type tobacco	259 (3.9)	21 (0.3)	280 (1.9)	2.1 (1.9-2.3)
Total	6712 (44.7)	8294 (55.3)	15006 (100.0)	100.0 (-)
Alcohol use				
Non-drinker	5320 (79.3)	8267 (99.7)	13587 (90.5)	89.5 (89.0-89.9)
Experienced (1-2 time)	1059 (15.8)	19 (0.2)	1078 (7.2)	8.0 (7.6-8.4)
Limited time (for treatment)	14 (0.2)	2 (0.0)	16 (0.1)	0.1 (0.07-0.2)
Exdrinker	39 (0.6)	3 (0.0)	42 (0.3)	0.3 (0.2-0.4)
Drinker	280 (4.2)	3 (0.0)	283 (1.9)	2.1 (1.9-2.3)
Drug abuse				
Non-drug abusers	6355 (94.7)	8292 (100.0)	14647 (97.6)	97.3 (97.1-97.6)
Experienced	104 (1.5)	0 (0.0)	104 (0.7)	0.8 (0.7-0. 9)
Drug abusers	253 (3.8)	2 (0.0)	255 (1.7)	1.9 (1.7-2.1)

CI, confidence interval.

Table 2 shows differences in smoking status by the demographic and socioeconomic features of the respondents. Results showed significant differences in tobacco smoking by all the studied variables.

**Table 2:** Differences in tobacco smoking bydemographic and socio-economic status of the Azar cohort participants (n = 15006)

**Characteristics**	**Tobacco Smoking**					
	**Nonsmoker**	**Cigarette ex-smoker**	**Cigarette regular smoker**	**Heavy cigarette smoker**	**Using other type tobacco**	***P *** **value**
Gender						
Male (6712)	47.8	18.0	18.1	12.3	3.9	< 0.001
Female (8294)	98.8	0.5	0.4	0.1	0.3	
Age						
35-39 (2606)	78.8	3.2	10.5	4.1	3.5	< 0.001
40-44 (2651)	80.3	4.6	8.1	4.7	2.2	
45-49 (2682)	76.6	7.3	8.7	6.1	1.2	
50-54 (2448)	70.8	11.5	8.8	8.0	0.9	
55-59 (2084)	72.8	11.2	8.5	6.3	1.2	
60-64 (1525)	75.1	12.7	5.9	4.3	1.9	
65-70 (1101)	76.1	12.6	5.1	4.0	2.2	
Socio-economic status						
Very low (2852)	81.9	6.2	6.1	4.7	1.1	< 0.001
Low (2876)	77.4	7.5	7.4	6.4	1.3	
Middle (2919)	73.9	8.8	9.1	6.3	1.9	
High (3000)	73.6	9.3	9.4	5.6	2.1	
Very high (3264)	72.0	10.2	10.2	4.6	3.0	
Alcohol use						
Non-drinker	97.9	76.1	67.3	54.9	63.9	< 0.001
Experienced	1.7	16.6	26.7	32.2	29.3	
Limited time	0.1	0.4	0.1	0.1	0.4	
Ex-drinker	0.1	0.6	0.7	1.2	1.4	
Drinker	0.3	6.3	5.2	11.6	5.0	
Drug abuse						
Non-drug abusers	99.8	96.4	90.5	80.8	95.7	< 0.001
Experienced	0.1	1.4	2.6	4.2	2.5	
Drug abusers	0.1	2.2	6.9	14.9	1.8	

Table 3 elucidates differences in alcohol use and drug abuse by the demographic and socioeconomic features of the participants. As shown in the table, significant differences were found in both variables by all demographic and socioeconomic variables.

**Table 3:** Differences in alcohol use and drug abuse by demographic and socio-economic status of the Azar cohort participants (n = 15006)

**Characteristics**	**Alcohol use**						**Drug abuse**			
	**Non-drinker**	**Experienced **	**Limited time **	**Ex-drinker**	**Drinker**	***P*** ** value**	**Non-drug abusers**	**Experienced **	**Drug abusers **	***P*** ** value**
Gender										
Male	79.3	15.8	0.2	0.6	4.2	< 0.001	94.7	1.5	3.8	< 0.001
Female	99.7	0.2	0.0	0.0	0.0		100.0	0.0	0.0	
Age										
35-39	88.3	9.6	0.1	0.2	1.8	< 0.001	97.8	0.6	1.6	0.026
40-44	90.9	7.6	0.2	0.3	1.1		98.1	0.4	1.5	
45-49	90.8	7.0	0.1	0.3	1.9		97.4	0.9	1.8	
50-54	90.4	6.6	0.2	0.4	2.4		97.1	0.6	2.3	
55-59	90.9	7.2	0.0	0.2	1.7		97.2	1.1	1.8	
60-64	91.5	5.8	0.0	0.4	2.4		97.5	0.7	1.8	
65-70	92.7	4.5	0.1	0.1	2.6		98.5	0.8	0.6	
Socio-economic status										
Very low	95.2	3.9	0.0	0.2	0.6	< 0.001	98.1	0.5	1.4	< 0.001
Low	91.4	6.5	0.1	0.4	1.6		98.0	0.5	1.5	
Middle	89.1	8.1	0.1	0.1	2.5		96.5	1.2	2.3	
High	89.2	8.4	0.1	0.4	1.9		97.1	0.9	1.9	
Very high	87.0	9.6	0.2	0.3	2.8		98.1	0.4	1.5	
Drug abuse										
Non-drug abusers	99.3	84.1	100.0	88.1	67.8	< 0.001	-	-	-	-
Experienced	0.2	4.5	0.0	0.0	7.8		-	-	-	
Drug abusers	0.4	11.3	0.0	11.9	24.4		-	-	-	

 The results of Tables 2 and 3 indicate that tobacco smoking, alcohol use, and drug use strongly correlated with each other. A total of 11.6% of heavy smokers consumed alcohol, compared to only 0.3% of non-smokers. Also, in contrast to only 0.1% of non-smokers, 14.9% of heavy smokers used illicit drugs. Moreover, 24.4% of alcohol users also abused drugs, whereas drug abuse in non-alcohol users was only 0.4%.

## Discussion

 In the present study, detailed prevalence estimates were provided for smoking, alcohol use, and drug abuse among the respondents of the Azar Cohort Study. The highest standardized prevalence rate was for regular cigarette smoking, and the lowest was for drug abuse.

 The standardized prevalence of regular and heavy cigarette smoking in the present study was 9.3% and 6.2%, respectively. These results are similar to those reported by Zahirian et al^16^ and Moradinazar et al,^14^ whose study populations were similar in terms of age group (35-70 years). In the Global Burden of Disease (GBD) study conducted by Peacock et al in 2015, the worldwide prevalence of daily smoking among adults (over 15 years of age) was roughly 15.2%^19^; this figure excluded non-daily smokers and ex-smokers.

 The standardized prevalence of drug abuse and alcohol use in the present study was 1.9% and 2.1%, respectively—lower than the figures reported by Moradinazar et al (11.9% for drug abuse and 9% for alcohol use).^14^ In their study, Moradinazar et al classified those who have used drugs and alcohol more than once in their lifetime as drug and alcohol users, generating remarkably high prevalence rates for these behaviors. Also, the worldwide prevalence of alcohol use (in the last 30 days) among adults (over 15 years old) is estimated at 18.4%.^19^ In general, alcohol is consumed less in Iran than in Western countries due to legal restrictions and religious prohibition.

 According to the results of our study, all three risky behaviors were generally more common among men than women, which is consistent with the results of previous national and international studies.^8,20,21^

 Our study observed a significant difference in smoking, alcohol use, and drug abuse by age. The prevalence of regular cigarette smoking decreased with age. Heavy cigarette smoking peaked in the 45–60 age group. We also found the prevalence of ex-smokers to rise with age, meaning that people quit when aging, possibly due to being diagnosed with various diseases.^22^ In contrast, Zahirian et al found that the prevalence of smoking increased with age.^16^ Pedro et al delineated a similar pattern; the highest smoking prevalence in both genders was in people 55–64 years of age.^23^

 According to our results, the prevalence of alcohol use increased with age, peaking among those aged 65–70 years. According to the results of a national epidemiologic survey on alcohol and its related factors in the United States, the increasing trend of alcohol use in the ≥ 65 age group had a higher speed than those in the younger age groups.^24^ In this American survey, the rising trend in alcohol use among older adults was remarkable and unprecedented compared with the results of previous surveys. According to previous studies, the pattern of alcohol use in older adults is such that they use alcohol more often but in lower amounts than younger people.^25^ According to the study conducted by Calvo et al, alcohol use peaks at the age of 60 and then decreases with age, with this decrease gaining speed at older ages. Regarding the differences between countries in the trends of alcohol use, cultural values related to alcohol use, gender, and religion have been shown to play a stronger role than the level of development and the price of alcohol.^26^

 In the present study, the highest prevalence of drug abuse was among the age group of 50–54, falling to the minimum rate in the 65–70 age group. Similar to our findings, a study conducted in Hong Kong showed that drug abuse peaked in middle-aged individuals and then declined after the age of 60.^27^

 We found significant differences in smoking, alcohol use, and drug abuse by SES. The prevalence of regular smoking and alcohol use was higher in the middle and upper socioeconomic classes, possibly due to the price of alcoholic beverages and cigarettes.^28^ Glei and Weinstein also showed that income reduction could decrease substance abuse, and alcohol use might decrease during a downturn.^29^ According to Calvo et al, older adults who live in more developed countries with lower alcohol prices tend to use more alcohol at the age of 50 compared with those in less developed countries with higher alcohol prices.^26^ In contrast, Lawana and Booysen, in a study in South Africa, showed that alcohol use was higher among lower socioeconomic classes.^30^

 We found that the prevalence of drug abuse was at its highest in the middle socioeconomic class. Since higher education positively correlates with higher income and social status, drug abuse among more educated people is usually lower than in less educated people.^27^ Moradinazar et al found more drug abuse in lower socioeconomic classes.^14^ Similarly, Pedro et al^23^ found a significant relationship between smoking and low education and income.

 Our study observed a significant relationship between smoking and alcohol, smoking and drug abuse, and alcohol and drug abuse. About 11.6% of those who were smokers also consumed alcohol. About 15% of the smokers were also drug abusers, and 24.4% of those who consumed alcohol also used drugs. These findings are consistent with those reported in other studies. In the study conducted by Britton et al on smokers aged 18–64 years, those who consumed more alcohol were 2.94 times more likely to smoke compared with those who did not consume alcohol.^31^ In their study, smokers had a higher rate of alcohol use than non-smokers. Smith et al. also found associations between smoking and alcohol use.^32^

 The strengths of the present study were the large sample size and high accuracy in data classification. However, there were some limitations. Self-reporting of data may have resulted in recall bias. Also, the age range of our participants was 35–70, which limits the comparison of our results with those of studies conducted on subjects aged above 15 years.

## Conclusion

 With a high level of accuracy in data classification, our study delineated the exact prevalence of the three risky behaviors in a large sample of the Iranian population. The prevalence of these behaviors was higher in men than women. We identified demographic and socioeconomic groups with a higher prevalence of all studied behaviors. Such high-risk groups should be targeted when designing substance abuse prevention programs. We also identified associations between the three risky behaviors, which shows the importance of simultaneously acting to minimize all three high-risk behaviors. Our results have implications for policy-makers wishing to design substance abuse prevention and cessation measures.

## Acknowledgements

 The authors would like to greatly acknowledge financial support for this study from Tabriz University of Medical Sciences. They also wish to thank all the participants of this study.

## Competing Interests

 The authors declare no competing interests.

## Ethical Approval

 This cross-sectional study was approved by Ethics Committee in Tabriz University of Medical Sciences (code: IR.TBZMED.REC.1401.099). All participants filled out and signed informed consent forms, and they had the right to leave the study whenever they want at any time.

## Funding

 This research study was funded by the Faculty of Health, Tabriz

 University of Medical Sciences.

## References

[1] Rafiee G, Ahmadi J, Rafiee F (2020). Prevalence of substance abuse (tobacco, alcohol, narcotics and psychotropic drugs) and its relationship to family factors in pre-university male students in Shiraz 2017-2018. J Community Health.

[2] Compton WM, Boyle M, Wargo E (2015). Prescription opioid abuse: problems and responses. Prev Med.

[3] World Health Organization (WHO). WHO Report on the Global Tobacco Epidemic, 2008: The MPOWER Package. Geneva: WHO; 2008.

[4] Nelson DE, Jarman DW, Rehm J, Greenfield TK, Rey G, Kerr WC (2013). Alcohol-attributable cancer deaths and years of potential life lost in the United States. Am J Public Health.

[5] Keethakumar A, Mehra VM, Khanlou N, Tamim H (2021). Cannabis use and patterns among middle and older aged Canadians prior to legalization: a sex-specific analysis of the Canadian Tobacco, Alcohol and Drugs Survey. BMC Public Health.

[6] World Health Organization (WHO). WHO Report on the Global Tobacco Epidemic, 2019: Offer Help to Quit Tobacco Use. WHO; 2019.

[7] Oh IH, Yoon SJ, Yoon TY, Choi JM, Choe BK, Kim EJ (2012). Health and economic burden of major cancers due to smoking in Korea. Asian Pac J Cancer Prev.

[8] Reitsma MB, Fullman N, Ng M, Salama JS, Abajobir A, Abate KH (2017). Smoking prevalence and attributable disease burden in 195 countries and territories, 1990-2015: a systematic analysis from the Global Burden of Disease Study 2015. Lancet.

[9] Bagnardi V, Blangiardo M, La Vecchia C, Corrao G (2001). Alcohol consumption and the risk of cancer: a meta-analysis. Alcohol Res Health.

[10] Hasan A, von Keller R, Friemel CM, Hall W, Schneider M, Koethe D (2020). Cannabis use and psychosis: a review of reviews. Eur Arch Psychiatry Clin Neurosci.

[11] Yayan J, Rasche K (2016). Damaging effects of cannabis use on the lungs. Adv Exp Med Biol.

[12] Hu Y, van Lenthe FJ, Platt S, Bosdriesz JR, Lahelma E, Menvielle G (2017). The impact of tobacco control policies on smoking among socioeconomic groups in nine European countries, 1990-2007. Nicotine Tob Res.

[13] Teimourpour A, Farzadfar F, Yaseri M, Hosseini M (2020). Spatial Survival analysis of initiation age and prevalence of smoking in Iran; results from a population based study. Arch Iran Med.

[14] Moradinazar M, Najafi F, Jalilian F, Pasdar Y, Hamzeh B, Shakiba E (2020). Prevalence of drug use, alcohol consumption, cigarette smoking and measure of socioeconomic-related inequalities of drug use among Iranian people: findings from a national survey. Subst Abuse Treat Prev Policy.

[15] Amirabadizadeh A, Nezami H, Vaughn MG, Nakhaee S, Mehrpour O (2018). Identifying risk factors for drug use in an Iranian treatment sample: a prediction approach using decision trees. Subst Use Misuse.

[16] Zahirian Moghadam T, Zandian H, Pourfarzi F, Poustchi H (2021). Environmental and economics-related factors of smoking among Iranian adults aged 35-70: a PERSIAN cohort-based cross-sectional study. Environ Sci Pollut Res Int.

[17] Farhang S, Faramarzi E, Amini Sani N, Poustchi H, Ostadrahimi A, Alizadeh BZ (2019). Cohort profile: the AZAR cohort, a health-oriented research model in areas of major environmental change in Central Asia. Int J Epidemiol.

[18] Ostadrahimi A, Nikniaz Z, Faramarzi E, Mohammadpoorasl A, Ansarin K, Somi MH (2018). Does long sleep duration increase risk of metabolic syndrome in Azar cohort study population?. Health Promot Perspect.

[19] Peacock A, Leung J, Larney S, Colledge S, Hickman M, Rehm J (2018). Global statistics on alcohol, tobacco and illicit drug use: 2017 status report. Addiction.

[20] Mohebbi E, Haghdoost AA, Noroozi A, Molavi Vardanjani H, Hajebi A, Nikbakht R (2019). Awareness and attitude towards opioid and stimulant use and lifetime prevalence of the drugs: a study in 5 large cities of Iran. Int J Health Policy Manag.

[21] Kritsotakis G, Psarrou M, Vassilaki M, Androulaki Z, Philalithis AE (2016). Gender differences in the prevalence and clustering of multiple health risk behaviours in young adults. J Adv Nurs.

[22] Qiu D, Chen T, Liu T, Song F (2020). Smoking cessation and related factors in middle-aged and older Chinese adults: evidence from a longitudinal study. PLoS One.

[23] Pedro JM, Brito M, Barros H (2017). Tobacco consumption and nicotine dependence in Bengo province, Angola: a community-based survey. PLoS One.

[24] Grant BF, Chou SP, Saha TD, Pickering RP, Kerridge BT, Ruan WJ (2017). Prevalence of 12-month alcohol use, high-risk drinking, and DSM-IV alcohol use disorder in the United States, 2001-2002 to 2012-2013: results from the National Epidemiologic Survey on Alcohol and Related Conditions. JAMA Psychiatry.

[25] Satre DD, Knight BG (2001). Alcohol expectancies and their relationship to alcohol use: age and sex differences. Aging Ment Health.

[26] Calvo E, Allel K, Staudinger UM, Castillo-Carniglia A, Medina JT, Keyes KM (2021). Cross-country differences in age trends in alcohol consumption among older adults: a cross-sectional study of individuals aged 50 years and older in 22 countries. Addiction.

[27] Liu T, Gietel-Basten S (2019). The demography of drug abuse in Hong Kong. China J Soc Work.

[28] Hiscock R, Bauld L, Amos A, Fidler JA, Munafò M (2012). Socioeconomic status and smoking: a review. Ann N Y Acad Sci.

[29] Glei DA, Weinstein M (2019). Drug and alcohol abuse: the role of economic insecurity. Am J Health Behav.

[30] Lawana N, Booysen F (2018). Decomposing socioeconomic inequalities in alcohol use by men living in South African urban informal settlements. BMC Public Health.

[31] Britton M, Derrick JL, Shepherd JM, Haddad S, Garey L, Viana AG (2021). Associations between alcohol consumption and smoking variables among Latinx daily smokers. Addict Behav.

[32] Smith CL, Jenkins G, Burduli E, Tham P, Miguel A, Roll J (2020). Crossover associations of alcohol and smoking, craving and biochemically verified alcohol and nicotine use in heavy drinking smokers. Behav Pharmacol.

